# Necrotising fasciitis: a case series set in rural Australia

**DOI:** 10.1093/jscr/rjad031

**Published:** 2023-02-07

**Authors:** Tegan J Kay, Lauren Wallace, Laura Bennett, Peter J Gallagher

**Affiliations:** Department of General Surgery, Wimmera Health Care Group, Horsham, Australia; Department of General Surgery, Wimmera Health Care Group, Horsham, Australia; Department of General Surgery, Wimmera Health Care Group, Horsham, Australia; Department of General Surgery, Wimmera Health Care Group, Horsham, Australia

## Abstract

Necrotising fasciitis (NF) is a rapidly progressive infection of soft tissue and fascia. Early diagnosis and prompt extensive surgical debridement decrease mortality. This remains a challenge for rural surgeons who have limited experience with the disease, in a setting where patient transfers to tertiary centres are lengthy and often delayed. To assist clinical decision making in this setting, a case series of five NF presentations in a rural Australian hospital were retrospectively analysed for presentation, investigation, treatment and clinical outcomes. Three underwent abdominal wall debridement and two underwent below knee amputation. Results demonstrate early recognition of NF and the extent of surgical intervention prior to acute transfer are key to successful outcomes. Expedient diagnosis and early extensive debridement at the initial contact reduce mortality and should be the goal of management in this setting.

## INTRODUCTION

Necrotising fasciitis (NF) is a rapidly progressive infection causing destruction of soft tissue and fascia. Cases are rare with an incidence of 1 per 100 000 [1] and mortality risk as high as 26% [2]. The majority of infections are polymicrobial [3] and most commonly affect the extremities, perineum and trunk [4]. Early infections present with localized, soft tissue symptoms [5], however left untreated by local debridement, these rapidly escalate to systemic deterioration and septic shock [6]. Early treatment involves broad-spectrum antibiotics and intensive care admission; however, the gold standard of management remains early and aggressive surgical debridement [6, 7].

Delay to diagnosis and surgical intervention increases the mortality of NF [8, 9]. It follows that NF is a challenging condition for rural surgeons due to unfamiliarity with the condition and a lack of resources preventing expedient operative intervention and transfer [9, 10]. Once NF is suspected, transfer to a tertiary referral centre should be initiated. Due to delays in this process and the inherent rapid progression of NF, urgent surgical intervention prior to transfer is required. Early and extensive debridement of all involved tissue significantly improves mortality compared with multiple, smaller operations and should be the aim of initial surgery [11]. However, due to inexperience with the disease, limited surgical debridement may occur rurally leaving diseased tissue *in situ* and driving ongoing sepsis during the transfer process. Limited literature exists describing the experience of NF in a rural setting to assist surgeons in this decision-making process. This case series set in rural Australia demonstrates successful outcomes of NF can be achieved through early diagnosis and aggressive surgical treatment prior to definitive transfer.

## CASE SERIES

Five consecutive cases of NF admitted to a rural Australian hospital within the last 12 months are presented below. Three involved the abdominal wall and two the lower limb. Clinical presentation and surgical outcomes are summarized in Tables 1 and 2, respectively. Average time to surgical referral and operative intervention were 2.2 and 7.2 h, respectively.

**Table 1:** Initial clinical presentation and investigations for Cases 1–5

	Case 1	Case 2	Case 3	Case 4	Case 5
**Clinical Presentation**	**Lower limb**		**Abdominal Wall**		
Age (years) and gender (male/female)	62 M	75 M	70F	60 M	51 M
Location of NF	Left lower limb	Left lower limb	Abdominal wall	Abdominal wall	Abdominal wall
Duration of symptoms prior to presentation	2 weeks	5 days	3 weeks	2 weeks	4 days
Previous GP/ED presentation symptoms?	Yes (GP)	Yes (GP)	Yes (ED)	Yes (GP/ED)	Yes (GP)
Features of sepsis on arrival to ED? (yes/no)	No	No	Yes	No	No
History of T2DM? (Hba1C)	Yes (10.3)	Yes (8.3)	Yes (unknown)	Yes (10.3)	Yes (10.8)
History of obesity? (weight)	Yes (114 kg)	Yes (108 kg)	Yes (112 kg)	No	Yes (136 kg)
LRINEC score^a^	4	9	8	1	1
**Initial pathology**					
WCC (neutrophils)	24.2 (21.0)	19.7 (18.3)	17.3 (14.3)	10 (7.8)	7.0 (4.5)
CRP	96.9	382	489.9	89.6	22
Lactate on admission	3.8	1.9	9.9	1.0	1.0
**Radiological investigation**					
Type of radiological investigation	CT left lower leg	X-ray left foot	CT abdomen/pelvis	CT abdomen/pelvis	CT abdomen/pelvis
Key imaging result	OM of great toe, extensive oedema and fat stranding to mid femur with subcutaneous gas to mid-tibia.	Subcutaneous gas within the subcutaneous soft tissues of the midfoot with marked soft tissue swelling.	Large volume abdominal wall fluid collection associated with extensive gas content.	Diffuse inflammation of the abdominal wall with gas in fascial planes and superficial skin ulceration.	Fat stranding of skin and superficial fascia with gas locules extending to base of the scrotum.

^a^Laboratory Risk Indicator for Necrotising Fasciitis (LRINEC) [12]

**Table 2:** Local surgical intervention and transfer details for Cases 1–5

	Case 1	Case 2	Case 3	Case 4	Case 5
**Operative intervention**					
Time to surgical referral from ED triage (hours)	2.0	2.0	1.0	5.0	1.0
Time to operative intervention from ED triage (hours)	3.25	2.5	5.0	19.0	6.0
Operation performed	Left BKA	Left BKA	Abdominal wall debridement	Abdominal wall debridement	Abdominal wall debridement
Microbiology	*Streptococcus agalactiae*, *S. anginosus* and Bacteroides.	*Escherichia coli*, *Klebsiella pneumoniae* and mixed anaerobes.	*E. coli*, *Morganella morganii*, *Enterococcus faecalis* and mixed anaerobes.	Mixed skin flora and anaerobes.	Numerous leukocytes but nil bacteria
**Clinical outcome**					
Transferred to tertiary centre (Y/N)	Y	Y	Y	Y	N
Number of further surgical debridements	1	0	5	0	0
Method of wound closure	Primary closure with staples	Primary closure with staples	Saline-soaked dressings then VAC dressing	Saline-soaked dressings then primary closure	Saline-soaked dressings then primary closure
Total days of admission at tertiary centre	11	23	45	27	NA

### Case 1 (Fig. 1)

A 62-year-old male presented with 2 weeks of left lower limb erythema and an ulcerated, necrotic great toe wound, initially treated as gout by his primary care provider. Computed tomography (CT) demonstrated features of NF to the level of mid-tibia and he underwent a left below knee amputation (BKA). He required only one further debridement following tertiary transfer and was transferred back for local rehabilitation 11 days later.

**Figure 1:**
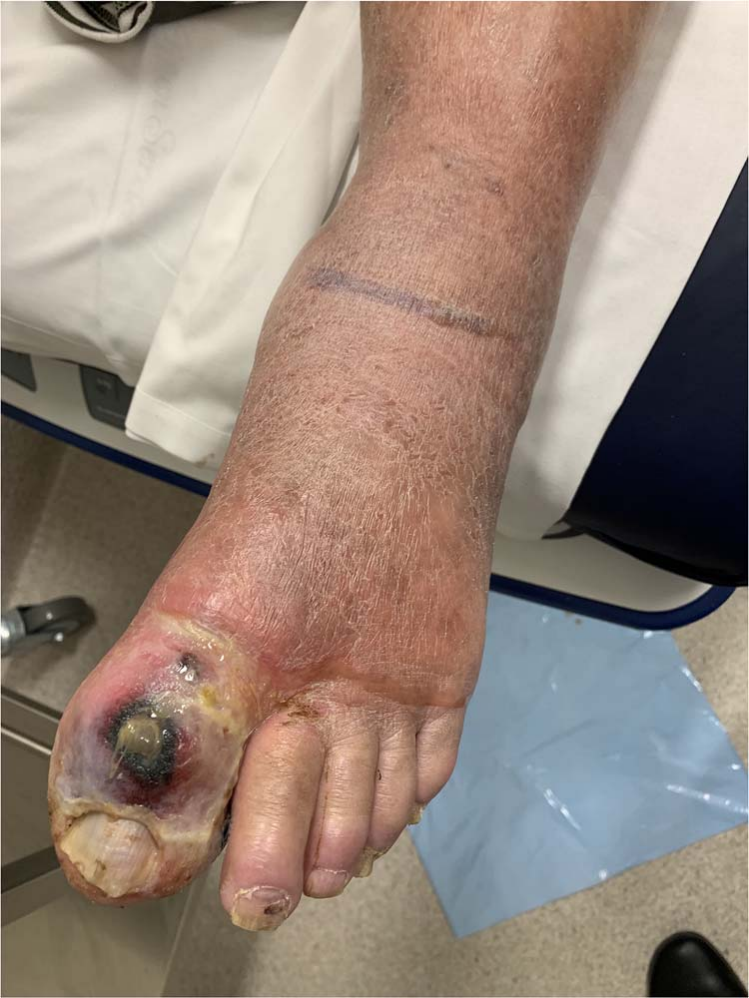
Clinical photography of the left foot of Case 1 at time of surgical referral.

### Case 2 (Fig. 2)

A 76-year-old male presented with 5 days of an increasingly painful, erythematous left foot, initially thought to be a superimposed superficial infection of gout. An X-ray demonstrated NF and he was taken to theatre for a left BKA. No further debridements occurred at the tertiary centre. He was transferred back for ongoing wound care and rehabilitation 23 days later.

**Figure 2:**
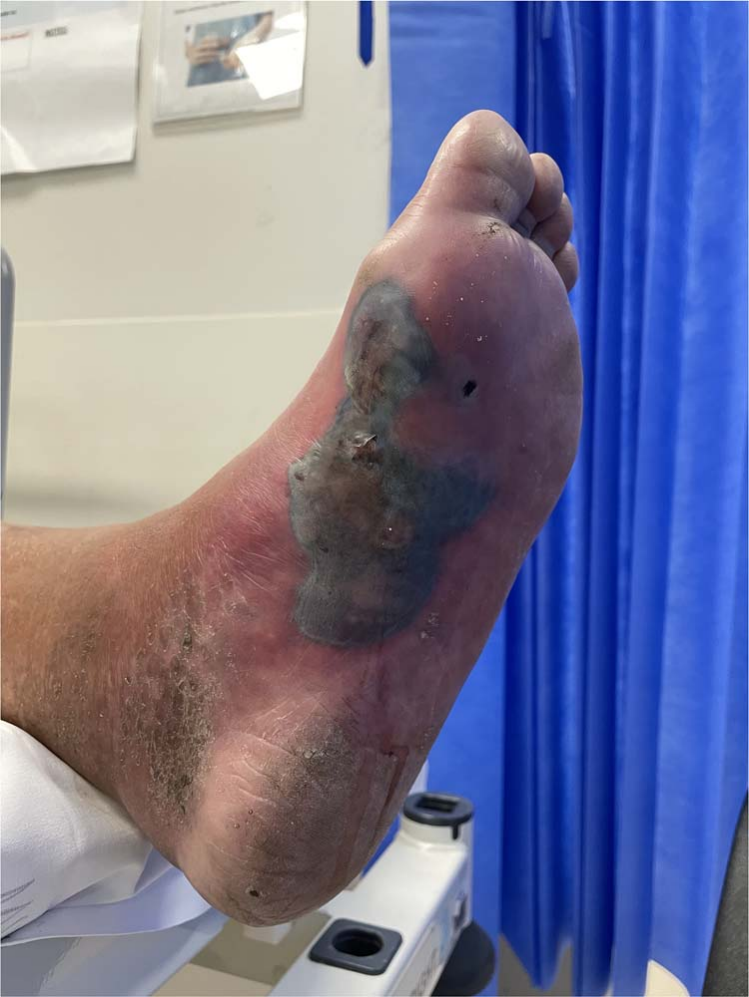
Clinical photography of the left foot of Case 2 at time of surgical referral.

### Case 3 (Fig. 3)

A 70-year-old female presented with a 3-week history of lower abdominal pain. Given clinically well with minimal symptoms, she was initially discharged home; however, represented 2 days later with sepsis and extensive necrosis of her abdominal wall. She was taken urgently for debridement and transferred to a tertiary centre. Five further debridements took place and she was transferred back for ongoing vacuum-assisted closure (VAC) dressings 45 days later.

**Figure 3:**
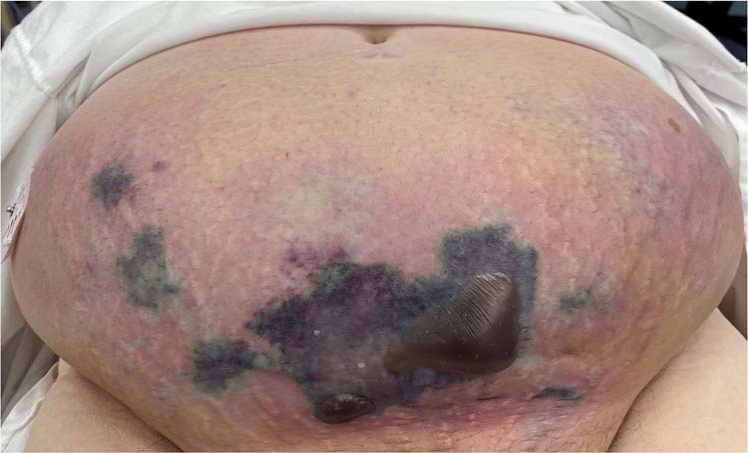
Clinical photography of the abdominal wall of Case 3 at time of surgical referral and intra-operatively.

### Case 4 (Fig. 4)

A 60-year-old male presented with an abdominal wall abscess, having previously been reviewed and discharged 1 week prior for vague abdominal pain associated with minor skin erythema. Initially, incision and drainage were performed under local anaesthesia. He became systemically unwell and a CT demonstrated abdominal wall NF. He proceeded to theatre for debridement. Primary closure occurred 1 month later and he was discharged directly home.

**Figure 4:**
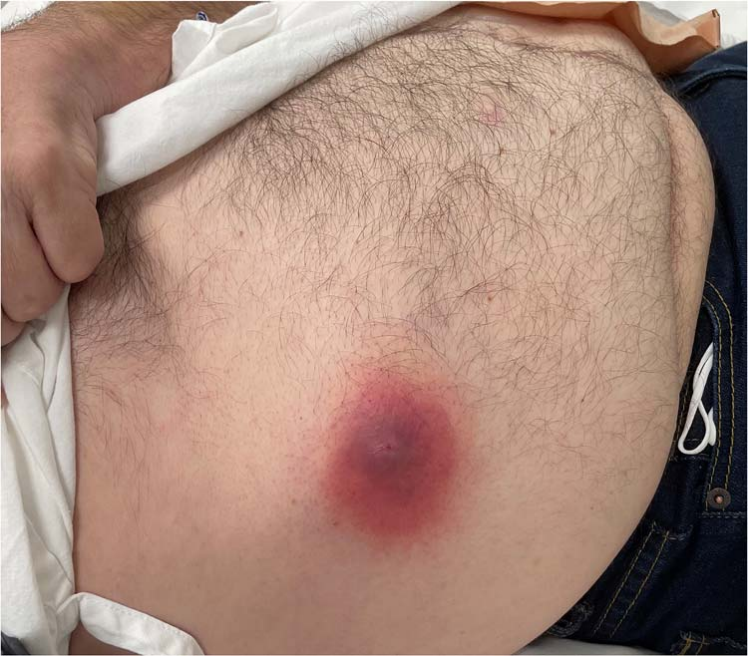
Clinical photography of the abdominal wall of Case 4 at time of surgical referral.

### Case 5 (Fig. 5)

A 51-year-old male presented to the ED with increasing abdominal wall erythema and pain following incision and drainage of a lower abdominal abscess 1 day prior in the surgical outpatient department. Although clinical suspicion for NF was low, a CT demonstrated abdominal wall NF and he was taken to theatre for debridement. The abdominal wall defect underwent primary closure 5 days later and he was discharged directly home Day 10 after presentation.

**Figure 5:**
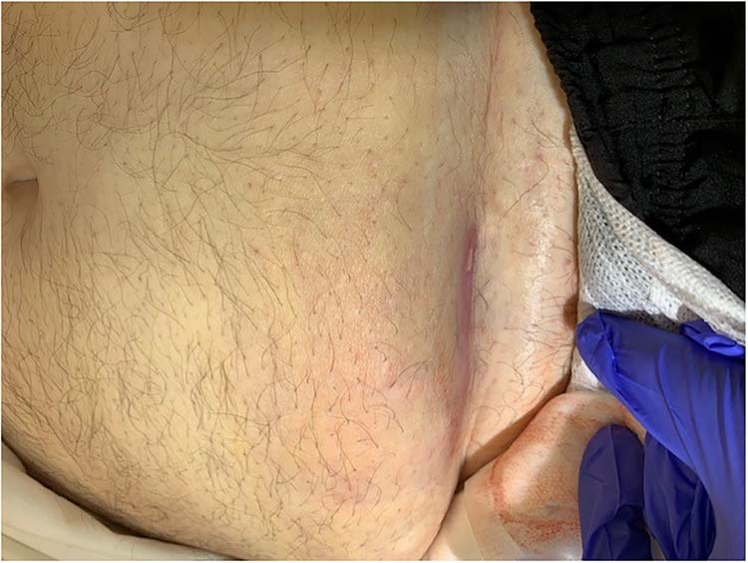
Clinical photography of the abdominal wall of Case 5 at time of surgical referral.

## DISCUSSION

NF is a serious, rapidly progressive soft tissue infection with Australian mortality rates as high as 18% [13]. Our review of five cases presenting over a single year reflects the rising incidence of NF globally, likely secondary to the worsening of chronic medical conditions during the Covid-19 pandemic [14]. Early recognition and early extensive debridement are paramount to successful outcomes. This is challenging for rural surgeons where time to definitive transfer is lengthy and patients often present late with features of septic shock. We present a spectrum of severity of NF on the torso or leg with all patients undergoing aggressive surgical intervention locally prior to tertiary transfer. This case series demonstrates the challenges of treating NF in the rural context and highlights successful patient outcomes with no mortality can be achieved through early recognition and extensive definitive debridement prior to transfer. We discuss two main challenges in the rural setting: time to diagnosis and the timing and extent of operative intervention.

### Time to diagnosis

Delayed recognition of NF significantly increases mortality [11, 15, 16]. However, diagnosis and clinical presentation may be difficult to distinguish from other less severe soft tissue infections [5]. This delays hospital presentation with rural patients experiencing symptoms on average 6 days prior to admission [10]. In this case series, three patients had symptoms for over 2 weeks and were discharged from ED prior to representing with more severe symptoms. These cases reflect a lesser known entity of sub-acute NF, which presents with slow symptom onset that unsuspecting clinicians may not recognize, causing a significant delay in diagnosis [8]. Suspicion should be heightened when cases have diabetes mellitus (DM) or obesity, the most common risk factors for NF in Australia [9]. All patients in this series had DM and the majority were overweight, increasing clinical suspicion for NF at initial review by the surgical team.

In an attempt to improve outcomes of NF, a recent study investigated delays to diagnosis and treatment using data from The Australian and New Zealand Audit of Surgical Mortality [9]. They found only 14% of patients were initially given the correct diagnosis of NF in ED and cases were often only referred to the surgical team once systemic symptoms developed [9]. This was exacerbated in rural settings, attributed to underresourced departments with a higher proportion of junior staff [9]. From a surgical perspective, diagnostic delay was most often associated with trainee surgical personnel with limited experience of NF [9]. This case series demonstrated an average time from ED triage to surgical referral of 2.2 h, illustrating the need for increased education and clinical suspicion of NF in the ED setting. To assist clinicians, the Laboratory Risk Indicator for Necrotising Fasciitis (LRINEC) may be utilized to improve diagnostic suspicion with a score greater than 8 being strongly predictive of a necrotising soft tissue infection [12]. However, the LRINEC score should be interpreted cautiously, given a recent Australian Study found it had a sensitivity and specificity of only 60.5% and 78.5% in the rural setting, respectively [10]. Patients within this case series had scores ranging from 1 to 9, illustrating NF is ultimately a clinical diagnosis and must be managed as such until proven otherwise.

For ambivalent cases, imaging and surgical cut down with urgent gram stain may assist diagnosis [5]. CT features of subcutaneous emphysema and fascial thickening can be present; however, inflammatory changes can also be secondary to superficial infection resulting in a lack of specificity [17]. Magnetic resonance imaging (MRI) is superior and has a sensitivity of 90–100% for NF [6]. However, both are difficult to access in rural environments and may actually delay treatment [18]. Three patients in our series presented after-hours when CT required a radiographer to be recalled in and no MRI was available. When in doubt, the ‘finger test’ may be utilized by suspecting surgeons in lieu of radiographic evidence of NF [19]. A small incision is made so that subcutaneous tissues can be directly observed for signs of necrosis and digital exploration can be used to assess for fascial involvement [19]. Urgent gram stain and fresh frozen section can be sent from this procedure with expedient results allowing for informed decision-making regarding intervention [5]. However, this relies on having 24-h pathology which is often limited in the rural setting. Ultimately, in this context, NF remains a clinical diagnosis and urgent surgical intervention should occur even when the diagnosis is unable to be confirmed due to lack of resources.

### Timing and extent of operative intervention

Once the diagnosis of NF is made, the decision of timing and extent of operative debridement remains difficult. Many surgeons have limited experience with NF and although the temptation exists to transfer cases to tertiary centres for specialist intervention, delay to operative debridement increases mortality [2, 16, 20]. Interhospital transfer is associated with increased mortality [21] with a 9-fold increase in death if intervention is delayed more than 24 h [8]. Two recent studies in regional Australia have demonstrated time is an issue for rural patients with NF, with an average delay of 31.6 h for acute transfer to a central service [10] and 40 h to operative intervention [13]. Cases in this series were taken to theatre locally within an average time of 7.2 h from ED triage. All patients demonstrated rapid postoperative improvement with control of sepsis and were stable at the time of transfer.

Once the decision has been made to operate locally on suspected NF, rural surgeons must avoid being cautious when it comes to the degree of surgical debridement. Prompt and extensive initial debridement remains the cornerstone of treatment and should be the aim of the primary operation [6]. Early extensive debridement significantly improves mortality from 38 to 4.2% when compared with multiple, smaller operations [11] and mortality rates are up to 7.5 times higher in those not adequately debrided [22]. This saves multiple unnecessary surgeries with as many as 22.3% of patients undergoing amputation or limb disarticulation following failure of multiple debridements to control infection [23]. In patients with pre-existing medical conditions or ongoing features of sepsis, fewer surgeries with shorter recovery time are likely to be beneficial [24] and should be considered especially in patients with poor prognostic factors such as old age, peripheral vascular disease and DM [6]. Even if tissue appears normal, an additional 5–10 mm margin of healthy tissue should be excised to ensure complete source control [25]. In this case series, the decision was made for both cases of lower limb NF to undergo BKA due to the extent of necrosis. Although these decisions were challenging, both cases had early control of sepsis with minimal further surgical intervention once transferred to a tertiary centre.

Additionally, early intervention may reduce the need for tertiary transfer. Case 5 had few symptoms to suggest NF at presentation; however, early CT was performed allowing expedient diagnosis and time to operative intervention. Urgent initial surgery meant infection was limited and debridement was not required to be as extensive. He avoided transfer to a tertiary centre and further debridements, allowing primary closure of his abdominal wall and discharged only 10 days after his initial presentation.

## CONCLUSION

NF remains a challenging condition for surgical teams in the rural setting. Lack of recognition, the dilemma surrounding local versus tertiary operative intervention and the extent of debridement are especially difficult for surgeons inexperienced with the disease. However, as this case series demonstrates, limb and lifesaving interventions can be performed in the rural setting with good outcomes and limited mortality. Supported by the literature, early diagnosis and expedient extensive debridement prior to transfer should be the goal of management in this context. Ultimately, this case series serves as a reminder that NF remains a clinical diagnosis and surgical teams must have a high index of suspicion with a low threshold for urgent, extensive surgical debridement to reduce morbidity and mortality rates in the rural setting.
